# Effects of blood flow restriction on mechanical properties of the rectus femoris muscle at rest

**DOI:** 10.3389/fphys.2023.1244376

**Published:** 2023-08-17

**Authors:** Jakub Jarosz, Dawid Gaweł, Michal Krzysztofik, Adam Zając, Athanasios Tsoukos, Gregory C. Bogdanis, Michal Wilk

**Affiliations:** ^1^ Institute of Sport Sciences, The Jerzy Kukuczka Academy of Physical Education, Katowice, Poland; ^2^ School of Physical Education and Sport Science, National and Kapodistrian University of Athens, Athens, Greece

**Keywords:** ischemia, reperfusion, myotonometric assessment, myotonometer, occlusion, stiffness, intramuscular pressure

## Abstract

**Introduction:** This study examined the effects of blood flow restriction (BFR) and reperfusion on the mechanical properties of the rectus femoris muscle at rest (frequency and stiffness).

**Methods:** Fourteen trained men (body weight = 81.0 ± 10.3 kg; BMI = 25 ± 3.0 m/kg^2^; height = 181 ± 4 cm; training experience = 6.0 ± 2.2 years) participated in an experimental session involving their dominant (BFR) and non-dominant leg (control). Muscle mechanical properties were measured using Myoton’s accelerometer at the midpoint of the rectus femoris muscle at five time points. In the BFR leg, an 80% arterial occlusion pressure was applied by a cuff for 5 min. No cuff was applied in the control leg. Femoral Myoton measurements were taken from both legs 2 and 4 min after the start of BRF as well as 30 s and 2 min after the end of the occlusion period.

**Results:** The two-way ANOVA revealed a statistically significant interaction effect for stiffness and frequency (*p* < 0.001; η^2^ > 0.67). The post hoc analysis showed that both stiffness and frequency increased during BFR compared with rest and then dropped to the resting levels post BFR period. Also, stiffness and frequency were higher than control only during the BFR period, and similar during rest and post BFR.

**Conclusion:** These results indicate that the application of BFR at rest leads to significant changes in mechanical properties of the rectus femoris muscle.

## Introduction

The objective of blood flow restriction (BFR) is to reduce blood flow to the muscles using an inflatable cuff or tourniquet applied around proximal parts of the upper or lower limbs (Loenneke et al., 2012). Cuffs that induce BFR may be readily used in various populations, including healthy, previously injured as well as patients with various heart conditions (Abe et al., 2005; Marocolo et al., 2018; Cahalin et al., 2022; Salagas et al., 2022). BFR training methods differ from each other, in terms of the aim and protocol used, e.g., pre-conditioning BFR performed before exercise to improve performance (Incognito et al., 2016; Marocolo et al., 2018; Wilk et al., 2020; Salagas et al., 2022), intermittent BFR during exercise (Gepfert et al., 2020), continuous BFR throughout the duration of resistance exercise (Takarada et al., 2000; Takarada et al., 2002; Wernbom et al., 2009; Patterson et al., 2019) or intra-conditioning BFR, i.e., only during the rest intervals of a series of exercise sets (Wilk et al., 2021a; Jarosz et al., 2021). These different BFR protocols may have an acute impact on post-exercise performance or muscle hypertrophy effects, with or without resistance exercise (Loenneke et al., 2012). Although the mechanisms underlying its effects have not been fully explored (Kubota et al., 2008), the phenomena initiating a cascade of physiological responses are triggered by the pooling of blood and fluid distal to the cuff, which enhances hydrostatic and osmotic gradients and finally increases intramuscular pressure and muscle volume (Jessee et al., 2018).

Intramuscular fluid pressure may have an effect on mechanical properties of muscle, which can be quantified by myotonometry (Korhonen et al., 2005). This method involves applying a small amount of force and evaluating the muscle’s response to it. The stiffness and frequency of oscillation indicate the level of tissue tone, with higher levels, indicating greater muscle tone (Alaca and Kablan, 2021). Intramuscular fluid pressure can have a significant impact on muscle function, affecting the geometry of muscle fibers and, as a result, the level of produced force (Sejersted and Hargens, 1995). Therefore, according to Konrad and Paternoster. (2022), evaluating this variable could contribute to the prediction of changes in muscle performance (Wang et al., 2017; Wang et al., 2017; Klich et al., 2019; Klich et al., 2020). For example, Klich et al. (2020) reported an acute increase in vastus lateralis stiffness after all-out 200 m and 4000 m track cycling, with a greater increase after the shorter sprint. Additionally, Trybulski et al. (2022a) observed a tendency for increased muscle stiffness of the triceps brachii long head with a simultaneous decrease in barbell velocity during the bench press exercise. The observed fatigue may be related to higher intramuscular fluid pressure which may impair clearance of metabolic byproducts. On the other hand, Krzysztofik et al. (2023) showed a decrease in vastus lateralis stiffness simultaneously with countermovement jump height improvements after low-volume and high-loaded back squats. This could partially explain the improvement in power or muscle strength reported previously after BFR application (Jarosz et al., 2021; Wilk et al., 2021). It seems that the increase in intramuscular fluid pressure induced by BFR applied at rest immediately before exercise could positively affect muscle metabolism (Murry et al., 1990; Andreas et al., 2011), increase blood flow to the muscles (Cunniffe et al., 2017), enhance neural activation (Cruz et al., 2017), and finally, as suggested by Kocman et al. (2015) allow the muscles to become more resistant to BFR and its potential deleterious effects during exercise.

To the best of the authors’ knowledge, no study to date has evaluated the acute effect of BFR at rest and after its cessation on changes in muscle mechanical properties using myotonometry. Since alterations in muscle mechanical properties, i.e., due to changes in intramuscular fluid pressure, might provide insight into muscle force production ability, hence this assessment could be used to determine the time required for reperfusion after BFR to limit the impact of high intramuscular fluid pressure on subsequent performance. Therefore, the purpose of this study was to examine changes in mechanical properties (frequency and stiffness) of the rectus femoris muscle during BFR as well as during reperfusion at rest. Since the enhancement in power output was previously observed immediately after the removal of BFR (Wilk et al., 2021a; Jarosz et al., 2021), it was hypothesized that during BFR muscle stiffness and muscle tone would significantly increase, and that immediately after removal of the cuff, they would return to baseline values.

## Materials and methods

### Study design

The aim of this study was to examine changes in mechanical properties of the rectus femoris muscle, namely: tone [oscillation frequency (Hz) and stiffness (N/m), under BFR without any physical activity]. Muscle properties of the rectus femoris of both legs were measured using the Myoton device, at 5 time points: at rest, 2 min and 4 min after the start of BFR, and 30 s and 2 min during reperfusion, after the release of BFR. BFR was applied for 5 min on the dominant lower limb with 80% of the total arterial occlusion pressure (AOP), while measurements of mechanical properties were also performed at the same time-points on the non-dominant limb without BFR (Control).

### Participants

Fourteen trained males participated in the study (body mass = 81.0 ± 10.3 kg; BMI = 25 ± 3.0 m/kg^2^; height = 181.0 ± 4.3 cm; training experience = 6.0 ± 2.2 years). The inclusion criteria were: a) age 18–40; b) resistance training at least 3 times a week, for at least 5 years; c) no cardiovascular diseases, e.g., thrombosis; d) no muscle injuries (leading to absence from training for more than 4 weeks) for at least 6 months before the start of the study. Participants were informed about the potential risks and benefits of participating in the project and about their right to withdraw from the study at any time, without providing any explanation for their decision. The participants signed a written consent to participate in the study but did not receive information about the purpose of the study and the expected results. All stages of the research were carried out at the Academy of Physical Education in Katowice, Poland. The experimental project was approved by the Bioethics Committee for Scientific Research (02/2019) at the Academy of Physical Education in Katowice, Poland, in accordance with the ethical standards of the Declaration of Helsinki, 1983. No participants withdrew from the study.

### Familiarization session

One week before the main experiment, the participants took part in a familiarization session. The familiarization session was performed to minimize possible learning effects during the main testing sessions and ensuring that the participants were well-prepared and accustomed to the tasks they were required to perform. During the familiarization session, measurements of frequency and stiffness, were performed at the middle point on the rectus femoris of both legs. The exact site of measurement was marked so as to perform all subsequent evaluations in the same position. For the BFR, the cuff was applied to the most proximal part of the dominant limb, while the cuff was not used for the control leg. The dominant leg was defined as the leg that the participants identified as their preferred or stronger leg for activities requiring single-leg performance (self-declaration). The familiarization protocol consisted of 3 min BFR with 2 min of reperfusion using a pressure of 100 mmHg.

### Experimental sessions

The participants were asked not to perform any resistance exercise 24 h before the start of the experimental session. They were also instructed to maintain their eating habits and not to use any supplements or stimulants before and during the week preceding the experiment. At the start of the experimental session body composition was evaluated using multi-channel bioelectrical impedance analysis in a laboratory environment with the InBody 370 device (InBody, Seoul, South Korea). Thereafter, the individual pressure of 100% AOP of the dominant lower limb was determined, and 80% AOP was used in the subsequent experimental protocol. Five minutes after determining the individual AOP, the mechanical properties of the rectus femoris muscle of both legs (BFR and control) were evaluated using the Myoton device. Measurements were taken at the middle of the length of the muscle (Muckelt et al., 2022), as previously determined. Immediately after the Myoton measurement, the cuff was applied on the dominant leg and was inflated to 80% of the individual AOP for 5 min. No cuff was applied on the other leg. Subsequent Myoton measurements of the rectus femoris muscle of both legs were performed 2 and 4 min after the cuff was inflated. The cuff was removed from the dominant leg after 5 min of occlusion, and further measurements were performed in both legs 30 s and 2 min after the end of BFR (reperfusion). During the entire duration of the evaluations the participants were still in the supine position with a foam roller under their knees to keep them slightly flexed.

### Blood flow restriction

A cuff (FitCuffs^®^, cuff width 10,5 cm, Denmark) was placed in the most proximal part of the dominant limb in order to determine the AOP of each participant (∼80% AOP conditions). After completing the general warm-up and a 5-min rest interval, the full occlusion arterial pressure (100% AOP) was determined (in a seated position). A handheld Edan SD3 Doppler with an OLED screen and a 2 MHz probe from Edan Instruments (Shenzhen, China) was used (Jarosz et al., 2021; Wilk et al., 2021). The AOP was measured twice and the measurements were 5 min apart in the subject Sieljacks et al. (2018), in case the obtained differences were within 20 mmHg, then the mean of the two measurements was used. The average 80%AOP used for BFR was (147 ± 24 mmHg).

### Measurement of muscle mechanical properties

MyotonPRO, is a non-invasive device monitoring superficial mechanical deformation of soft tissues (MyotonPRO, Myoton AS, Tallinn, Estonia), and was used to assess the mechanical properties of the rectus femoris muscle. Measurements were performed at the midpoint of the rectus femoris muscle build on previous research (Mullix et al., 2012; Agyapong-Badu et al., 2016; Gacto-Sánchez et al., 2023). The midpoint of the rectus femoris was determined in the supine position with a foam roller under the knee (Muckelt et al., 2022) with the aid of a tape measure applied on the line formed between the upper edge of the patella and the iliac spine of the pelvis. The rectus femoris muscle has been selected due to its crucial role in knee extension and hip flexion, and feasibility and accessibility for measurement (its superficial location in the thigh makes it more amenable to non-invasive assessment). The following muscle mechanical properties were measured: the Natural Oscillation Frequency [Hz], which is the intrinsic tension of the muscle in its passive state, characterizing tone or tension and the dynamic stiffness, which indicates the resistance to deformation (Salagas et al., 2022). Myoton’s accelerometer was set to 3200 Hz, and the average value kept for analysis was obtained from five consecutive measurements (0.4 N for 15 m) (Szymczyk et al., 2022).

### Statistical analyses

Data were analyzed using Statistica 9.1. The Shapiro-Wilk test was used to verify normality, while the homogeneity of variance was assessed by the Levene’s test. Statistical differences between BFR and control independently for frequency and stiffness were analyzed by two-way repeated measures ANOVA [(BFR vs. control leg) × 5 measurement time points]. The partial eta squared was used to determine effect sizes (ES). Partial eta squared values were classified 0.01–0.059 as small, 0.06–0.137 as moderate, and >0.137 as large. Post hoc comparisons using the Tukey’s test were conducted to locate the differences between mean values when a main effect or an interaction was found. For pairwise comparisons, ESs were determined by Cohen’s d which was characterized as trivial: d < 0.20; small: d between 0.20 and 0.49; moderate: d between 0.50 and 0.80, and large: d > 0.80. The statistical significance was accepted at *p* < 0.05.

## Results

Post-hoc power analysis using G*Power version 3.1.9.2 (Dusseldorf, Germany) for the parameters, such as “ANOVA, repeated measures, within factors,” was assumed as a statistical test (1 group of subjects, 2 experimental conditions, and 5 measurements) and the significance level of 0.05, indicated that effect size of at least 0.31 is needed to achieve a power above 80%. The two-way ANOVA showed a statistically significant interaction effect for stiffness (*p* < 0.001; η^2^ = 0.84) and frequency (*p* < 0.001; η^2^ = 0.81). The ANOVA also showed a statistically significant main effect of condition for stiffness (*p* < 0.001; η^2^ = 0.76) and frequency (*p* < 0.001; η^2^ = 0.60). The *post hoc* analysis for interaction showed that both stiffness and frequency increased during BFR compared with rest and then dropped to the resting levels during the reperfusion period. Also, stiffness and frequency were higher than control only during the BFR period, and similar during rest and post BFR (*p* < 0.001 for all, see Table 1).

**TABLE 1:** Muscle stiffness and frequency as determined by Myoton measurements in the blood flow restricted (BFR) and Control leg during BFR and reperfusion. ES: Cohen’s d effect size.

	Stiffness (N/m)				
		Blood flow restriction period		Reperfusion	
Condition	Rest	2 min	4 min	30 s	2 min
**BFR**	278.3 ± 15.7	409.9 ± 70.95^a^#	418.6 ± 63.9^a^#	280.1 ± 22.5	277.6 ± 20.7
**CONTROL**	279.4 ± 21.6	278.3 ± 18.6	282.3 ± 21.2	281.0 ± 22.3	280.0 ± 22.2
**ES**	0.06	2.54	2.87	0.04	0.11

	Frequency (Hz)				
		Blood flow restriction period		Reperfusion	
CONDITION	Rest	2 min	4 min	30 s	2 min
**BFR**	15.17 ± 0.92	19.56 ± 2.63^a^#	19.77 ± 2.40^a^#	15.22 ± 0.76	15.07 ± 1.01
**CONTROL**	15.31 ± 1.13	15.21 ± 1.05	15.44 ± 1.32	15.34 ± 1.31	15.54 ± 1.19
**ES**	0.14	2.17	2.24	0.11	0.43

^a^ 0.001 from control at the corresponding time point. #: 0.001 from Rest in the corresponding condition, *p* < 0.001.

## Discussion

The main finding of the study was that the application of BFR causes significant changes in mechanical properties of the rectus femoris muscle, i.e., stiffness and frequency, at rest. Specifically, a significant increase in stiffness and frequency were found only during the application of BFR compared to the control leg, but this effect disappeared immediately after the end of BFR. Furthermore, changes in mechanical properties of the studied muscle, were similar after 2 and 4 min of BFR, indicating that they were not dependent on the duration of BFR, at least for this BFR period. Additionally, the observed increases in frequency and stiffness of the rectus femoris muscle returned to baseline values very fast, i.e. 30 s after the removal of BFR. Therefore, the changes in mechanical properties of the muscle, as assessed by myotonometry last only during BFR and are quickly removed during the reperfusion period.

The increase in muscle stiffness and frequency observed in the BFR condition may be related to higher intracellular fluid levels and the associated increase in intracellular fluid pressure. Such an increase in intracellular fluid levels is one of the physiological factors arising from the accumulation of fluid distal to the cuff, increasing hydrostatic and osmotic gradients, and ultimately increasing muscle volume and intramuscular pressure (Jessee et al., 2018), which are related with changes in the mechanical properties of muscles. Previous studies examining changes in the level of intracellular fluids during exercise performed under BFR (Wilk et al., 2018) could not separate the effect of exercise *per se* from the effect of BFR on the mechanical properties of the muscles involved. The results of the present study indicate that an increase in muscle stiffness and frequency may be induced only from BFR at rest, thus separating the effects of exercise and BFR. Therefore, changes in the mechanical properties of muscles do not need to be exercise-related, as suggested by Hill et al. (2022). These authors emphasized that changes in mechanical properties of muscles are related to the type, intensity and volume of exercise, however, our study showed that such changes following BFR may be mainly related to the increase in intracellular fluid levels caused by external compression reducing blood flow. It should be noted that due to the constrictive nature of muscle contractions, the physiological effect of muscular contractions in combination with the exercise-induced plasma volume shifts to the working muscles during exercise, may also increase the level of intracellular fluids similar to that observed with resting BFR (Egan et al., 2006; Wilk et al., 2018; Wilk et al., 2021b). The results of the present study showed a significant increase in muscle stiffness and frequency during BFR compared to the control. Similar changes in stiffness and frequency to those observed in the present study, have been previously reported during exercise (Klich et al., 2019; Klich et al., 2020). According to Klich et al. (2020) the increase in the level of intracellular fluids during exercise reduces capillary blood flow, and causes a decrease in muscle strength, early muscle pain and fatigue, which may be associated with an increase in muscle stiffness (Wang et al., 2017; Wang et al., 2017; Klich et al., 2019; Klich et al., 2020; Trybulski et al., 2022b). It can therefore be postulated that the use of BFR at rest may induce similar changes in muscle mechanical properties to intense muscle actions during exercise.

Changes in mechanical properties of muscles were identical after 2 and 4 min of BFR. Thus, it may be suggested that the duration of BFR has no significant effect on the mechanical properties of muscles and the change in intracellular pressure, at least for the BFR period examined. The lack of significant changes in mechanical properties of muscles during the 2–4 min period under BFR may indicate a similar level of intracellular fluid pressure at these two time points. Maintaining constant values of intracellular pressure from the use of BFR may have a significant impact on the level of free radicals, lactate, hypoxia inducing factors (HIF) and heat shock proteins (HSP) in the resting muscle, and may contribute to the process of muscle activation and regeneration (Bemben et al., 2022).

In the present study, the mechanical properties of muscles returned to baseline values just 30 s after BFR removal. To the best of our knowledge, there is currently no evidence available to compare our study, especially in terms of reperfusion time and changes in muscle properties. The increased blood flow resulting from the use of BFR at rest may be related to the rapid return of mechanical properties of the muscles to resting values. Also, the rapid recovery of mechanical properties of the muscles to baseline values suggests that BFR at rest has a strong but short-term effect on quadriceps neuromuscular function. The benefits resulting from the direct return of mechanical properties of the muscles to baseline values observed in the present study confirm the results of Trybulski et al. (2022b), who argued that reperfusion time may also be a significant factor in BFR-induced acute adaptive changes.

The main limitation of the presented study is the lack of possibility to compare and generalize the results between different research protocols. As this is currently the only study analyzing the impact of BFR on changes in the mechanical properties of the rectus femoris muscle, further comparisons with other research protocols are not feasible. One methodological limitation is the absence of an analysis of the biochemical changes in the blood accompanying the application of BFR at rest. Such analysis could significantly influence the obtained results and provide a broader understanding of this topic. Another limitation of the present study is the lack of exercise performance measurements during and after BFR, so as to examine the changes in stiffness on muscle strength and power. However, previous studies Wilk et al. (2020) have shown improvements in peak bar velocity during bench press when performed under continuous BFR, and one possible mechanism contributing to this improvement may be an increase in stiffness. However, this remains to be directly examined in future studies.

## Conclusion

The results of this study indicate that the application of BFR at rest causes significant changes in mechanical properties of the rectus femoris muscle, i.e., stiffness and frequency. However, this effect is only observed during BFR and disappears immediately after removing BFR. This suggests that BFR increases stiffness and frequency of muscles only during its application, without affecting post-exercise mechanical properties, as assessed by myotonometry.

## Data Availability

The raw data supporting the conclusion of this article will be made available by the authors, without undue reservation.
